# Combinations of Histone Deacetylase Inhibitors with Distinct Latency Reversing Agents Variably Affect HIV Reactivation and Susceptibility to NK Cell-Mediated Killing of T Cells That Exit Viral Latency

**DOI:** 10.3390/ijms22136654

**Published:** 2021-06-22

**Authors:** Daniela A. Covino, Maria G. Desimio, Margherita Doria

**Affiliations:** Primary Immunodeficiency Research Unit, Bambino Gesù Children’s Hospital, IRCCS, 00165 Rome, Italy; danielaangela.covino@opbg.net (D.A.C.); mariagiovanna.dsm@gmail.com (M.G.D.)

**Keywords:** HIV-1 cure, shock-and-kill, latency reversing agents, histone deacetylase inhibitor, PKC agonist, proteasome inhibitor, Toll-like receptor agonist, NK cells, NKG2D, NKG2D ligand

## Abstract

The ‘shock-and-kill’ strategy to purge the latent HIV reservoir relies on latency-reversing agents (LRAs) to reactivate the provirus and subsequent immune-mediated killing of HIV-expressing cells. Yet, clinical trials employing histone deacetylase inhibitors (HDACis; Vorinostat, Romidepsin, Panobinostat) as LRAs failed to reduce the HIV reservoir size, stressing the need for more effective latency reversal strategies, such as 2-LRA combinations, and enhancement of the immune responses. Interestingly, several LRAs are employed to treat cancer because they up-modulate ligands for the NKG2D NK-cell activating receptor on tumor cells. Therefore, using in vitro T cell models of HIV latency and NK cells, we investigated the capacity of HDACis, either alone or combined with a distinct LRA, to potentiate the NKG2D/NKG2D ligands axis. While Bortezomib proteasome inhibitor was toxic for both T and NK cells, the GS-9620 TLR-7 agonist antagonized HIV reactivation and NKG2D ligand expression by HDACis. Conversely, co-administration of the Prostratin PKC agonist attenuated HDACi toxicity and, when combined with Romidepsin, stimulated HIV reactivation and further up-modulated NKG2D ligands on HIV^+^ T cells and NKG2D on NK cells, ultimately boosting NKG2D-mediated viral suppression by NK cells. These findings disclose limitations of LRA candidates and provide evidence that NK cell suppression of reactivated HIV may be modulated by specific 2-LRA combinations.

## 1. Introduction

The combinatorial antiretroviral therapy (ART) against HIV effectively suppresses viral replication but does not eliminate integrated provirus that persists in a small fraction of latently infected cells, mainly resting CD4^+^ T lymphocytes [1,2,3]. Hence, to prevent virus rebound and disease progression, HIV-infected patients must take ART for life, which can result in emergence of viral resistance, cumulative toxicity, chronic immune activation, and other morbidities.

In the last decade, major efforts have been made to identify strategies for eliminating HIV reservoirs through pharmacologic viral reactivation and killing of cells harbouring reactivated virus by a combination of host immune responses, viral cytopathic effects, and ART. For this approach, referred to as ‘shock-and-kill’, a plethora of compounds functioning as latency-reversing agents (LRAs) have been identified [4,5,6]. Reflecting the complexity of mechanisms that contribute to suppress HIV expression in latently infected cells (i.e., epigenetic, transcriptional, and post-transcriptional mechanisms) [7], candidate LRAs belong to distinct functional categories that include, but are not limited to: histone deacetylase inhibitors (HDACi), protein kinase C agonists (PKCa), Toll-like receptor agonists (TLRa), and activators of the PI3K/Akt pathway. Several LRAs have entered into HIV cure clinical trials though, at present, none has demonstrated clearance of latent infection. Specifically, administration to ART patients of an HDACi such as Vorinostat (VOR), Romidepsin (ROM), or Panobinostat (LBH589; PAN), of the PI3K/Akt activator Disulfiram, the PKCa Bryostatin (BRY), and the TLRa MGN1703 resulted in increased HIV RNA transcription but not in a reduction of the latent reservoir size [8,9,10,11,12,13,14,15,16]. Therefore, major efforts are being devoted to identify novel compounds with improved LRA efficacy as well as combinations of two functionally distinct LRAs that might synergize [6,17]. There is a growing evidence that HDACis enable HIV transcription initiation yet they do not remove other blocks impeding viral RNA elongation and splicing or production of late viral proteins [18,19], which may account for the clinical inefficacy of these drugs. However, synergistic in vitro or ex vivo HIV reactivation was found by combining HDACi with PKCas, which potently stimulate HIV transcription, or with proteasome inhibitors such as Bortezomib (BOR), which induces the viral Tat protein and stabilizes HIV transcription elongation complex [20,21,22,23,24,25,26].

Importantly, LRAs must be considered carefully not only for efficacy but also for the absence of negative effects on antiviral immune responses. Actually, some studies demonstrated that in vitro exposure to various HDACis and PKCas affected the viability and/or function of primary cytotoxic CD8^+^ T cells (CTLs) [27,28,29]. Similar studies on the impact of HDACis and PKCas on natural killer (NK) cells reported heterogeneous results, showing either immunosuppressive or stimulating effects [30,31,32,33,34,35]. Recent evidence suggests that NK cells have an important role in the containment of latent HIV reservoir. First, results from the VOR and PAN trials showed that the frequency and the function of NK cells, not CTLs, were the major correlates of the decrease in viral DNA levels in a group of ART patients [36,37]. In addition, it was shown that resting HIV-infected T cells, either generated by experimental cell models of latency or derived from ART patients, are efficiently killed by NK cells upon virus reactivation [32,35,38,39,40]. In general, NK cells possess a natural cytotoxic activity against virus-infected cells and tumors that is independent of antigen recognition, hence not affected by CTL escape mutations that typically accumulate within HIV provirus in ART patients and is regulated through the balance of opposing signals delivered by activating and inhibitory receptors [41].

In the context of NK-cell recognition and killing of HIV^+^ CD4^+^ T cells, the NKG2D activating receptor is engaged by cell-surface molecules (NKG2D ligands, NKG2DLs) that are induced during productive HIV infection in T cells, and delivers a potent stimulatory signal resulting in cytotoxicity [42,43,44,45,46]. The NKG2DLs belong to two family of proteins, the MHC-class-I-related sequence A and B (MICA, MICB) and the cytomegalovirus UL16-binding (ULBP1-6) proteins, whose expression is highly restricted in normal cells but can be induced in stressed cells, such as virus-infected or transformed cells, through epigenetic, transcriptional, and post-transcriptional mechanisms [47]. Of note, several LRAs originally entered into clinical trials to cure cancer patients because of their capacity to up-regulate NKG2DLs on tumor cells promoting their elimination by NK cells [48,49,50], suggesting that NKG2DL and latent HIV share common regulatory mechanisms. On the basis of the current knowledge, these mechanisms include, at least: epigenetic suppression, stimulation via the PI3K/Akt pathway and activation of transcription factors such as NF-κB and Sp1. Starting from these observations, we recently demonstrated that both VOR and the PKCa Prostratin (PRO) cooperated with HIV at up-modulating NKG2DLs, particularly ULBP2, on T cells that exit from viral latency and, as a consequence, become susceptible to NKG2D-mediated killing by NK cells [35,38]. We then proposed an approach to achieve eradication of HIV reservoirs through the employment of LRAs that effectively induce both NKG2DLs and latent provirus while boosting NKG2D-mediated cytotoxic responses of NK cells [51].

Pursuing this goal, in the present study we screened 2-LRA combinations for their capacity to reactivate HIV and up-regulate NKG2DL while preserving cell viability in experimental T cell models of latency. LRA combinations analyzed consisted in one leading HDACi candidate (VOR, PAN, ROM), associated with a functionally distinct LRA including: PRO, BOR, and the GS-9620 TLR-7 agonist [52,53,54] that is currently tested in ART patients. Selected combinations were then tested for their impact on NK cell viability, phenotype, and function. Finally, the ROM/PRO combination was evaluated for its effects in NK-cell mediated killing of autologous CD4^+^ T cells that exit HIV latency using a primary cell-based experimental system in which both effector and target cells were equally exposed to the drugs, showing enhanced NKG2D-mediated viral suppression.

## 2. Results

### 2.1. Two-Drug Combinations Variably Affect Expression of HIV and NKG2DLs in J1.1 Cells

**Figure 1:**
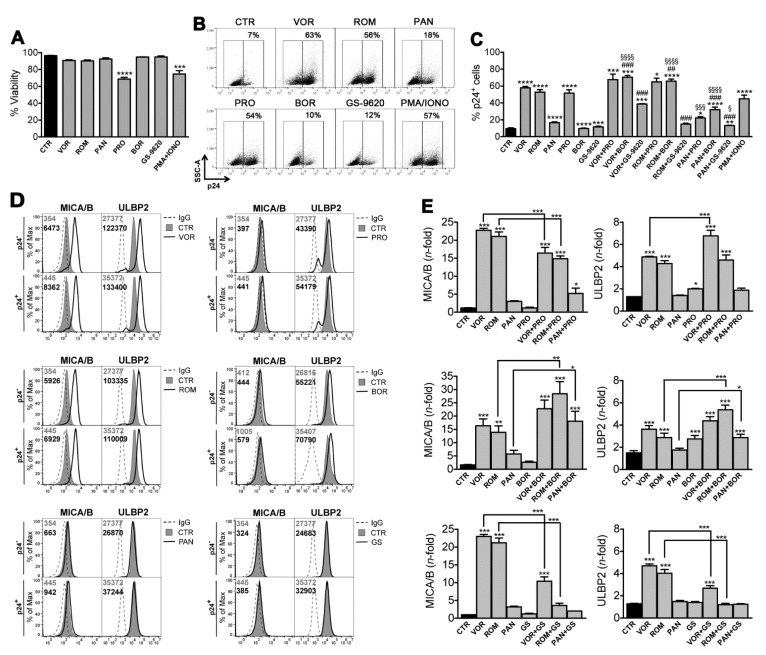
HIV-1 reactivation and modulation of NKG2DLs expression by two-drug combinations in J1.1 cells. J1.1 cells were treated for 48 h with solvent as control (CTR), 100 ng/mL PMA plus 1 μg/mL Ionomycin (PMA/IONO) as maximal stimulation, 10 µM VOR, 20 nM ROM, 20 nM PAN, 1 µM PRO, 5 nM BOR, or 3 µM GS-9620 used alone or combining each HDACi (VOR, ROM, PAN) with PRO, BOR, or GS-9620. Then, cells were analyzed for cell viability, HIV-1 reactivation (i.e., intracellular p24 expression) and cell-surface MICA/B and ULBP2 expression by multicolor flow cytometry. (**A**) The viability of treated J1.1 cells was examined by LIVE/DEAD staining and expressed relatively to CTR samples set at 100%. Bars represent mean ± SEM (*n* = 11). *** *p* < 0.001, **** *p* < 0.0001 by paired *t*-test. (**B**) Representative dot plots show the frequency of p24^+^ J1.1 cells with reactivated HIV following single treatments. (**C**) HIV reactivation quantified as %p24^+^ cells was determined in 11 independent experiments (mean ± SEM). Statistics was performed using paired *t*-test; versus control (CTR): * *p* < 0.05, ** *p* < 0.01, *** *p* < 0.001, **** *p* < 0.0001; versus HDACi alone: ^##^ *p* < 0.01, ^###^ *p* < 0.001; versus 2nd LRA alone: ^§^ *p* < 0.05, ^§§§^ *p* < 0.001, ^§§§§^ *p* < 0.0001. (**D**) Histograms show MICA/B and ULBP2 fluorescence in gated p24^-^ and p24^+^ cells in a representative experiment with J1.1 cells following the indicated treatments. Filled gray and open histograms represent staining of CTR and treated cells, respectively. Signal obtained with control IgG (dashed line) and mean fluorescence intensity (MFI) values for CTR (gray) and treated (black) cells are indicated. (**E**) Quantification of MICA/B (left panels) and ULBP2 (right panels) up-modulation induced by single or 2-drug combination on p24^+^ cells relative to p24^-^ CTR cells. Bars represent mean ± SEM (*n* = 7). Statistics was performed using One-way Anova with Bonferroni post test to perform multiple comparisons; significant differences versus control (CTR) and versus HDACi alone are indicated: * *p* < 0.05, ** *p* < 0.01, *** *p* < 0.001.

We used the J1.1 T cell line latently infected with HIV to investigate the simultaneous effect on virus reactivation and NKG2DLs expression of 2-drug combinations composed of one HDACi (VOR, ROM, PAN) and a mechanistically distinct LRA including a PKCa (PRO), a proteasome inhibitor (BOR), and a TLR-7 agonist (GS-9620). The drugs were added for 48 h to J1.1 cultures at concentrations that were previously shown to be effective at reactivating HIV-1 by in vitro or ex vivo latency systems (10 µM VOR, 20 nM ROM, 20 nM PAN, 1 µM PRO, 5 nM BOR, 3 µM GS-9620 [22,25,31,35,38,53,55]; as controls, J1.1 cells were cultivated in the presence of solvent alone (control, CTR) or maximally stimulated with 100 ng/mL PMA plus 1 μg/mL Ionomycin (PMA/IONO). In a preliminary assay, each tested LRA did not affect J1.1 cell viability with the exception of PRO and PMA/IONO (34% and 26% reduction, respectively; Figure 1A), an effect that is consistent with the overgrowth and exhaustion of cells exposed to these strong activating treatments. In line with previous data [25,38,53,56,57,58], we found that J1.1 cell exposure to each single drug reactivated latent HIV-1 above the level of spontaneous viral activation (8% of untreated cells expressing viral p24 Gag), yet to an extent that varied considerably: VOR, ROM, and PRO potently reactivated latent HIV alike PMA/IONO (up to 60% cells became p24^+^), whereas PAN had a lower activity (17% p24^+^) and BOR and GS-9620 had a negligible effect (9% and 11% p24^+^, respectively) (Figure 1B,C). We also found that, despite not reaching statistical significance, combining VOR or ROM with PRO consistently resulted in an increase of HIV reactivation if compared with each drug alone (Figure 1C); on the other hand, PAN/PRO combination resulted in 22% p24^+^ cells, hence in a reactivation that was modestly increased if compared to PAN-treated cells but drastically reduced if compared with cultures exposed to PRO alone. In addition, combining VOR, ROM, or PAN with BOR significantly increased the frequency of p24^+^ cells induced by each LRA used one at a time, indicating the existence of a general cooperative interaction between HDACis and BOR. Conversely, GS-9620 exerted an antagonist effect when combined with VOR, ROM, or PAN, resulting in a significant reduction of HDACi capacity to induce individually HIV-1 reactivation (Figure 1C).

In basal conditions, J1.1 cells express NKG2DLs (i.e., MICA, MICB, ULBP1, ULBP2), which is coherent with their leukemic origin and with low-level viral protein expression by latent HIV [38]. We previously showed that exposure of J1.1 cells to VOR increased *MICA, MICB,* and *ULBP2* transcription and, in reactivated p24^+^ cells, was associated with intracellular accumulation of newly synthesized ligands by yet unknown viral protein(s) [38]; as a result, cell-surface ligand up-regulation by VOR was similar in p24^-^ and p24^+^ cells (only MICB was significantly higher in p24^+^ cells) [38]. Here, the treated J1.1 cultures were analyzed for the expression MICA/B (using mAb cross-reacting with MICA and MICB proteins) and ULBP2 on both p24^−^ and p24^+^ cells as shown for a representative experiment in Figure 1D (complete data are shown in Supplementary Figure S1) and the extent of ligand modulation is reported for p24^+^ cells in Figure 1E. Results showed that, in agreement with NKG2DL up-modulation by HDACi reported in previous studies [49,50], VOR and ROM strongly enhanced MICA/B and ULBP2 expression on p24^-^ and, to somewhat higher level, on p24^+^ cells, yet PAN had only a minimal effect on MICA/B. Exposure to PRO or BOR was ineffective on MICA/B but both increased ULBP2 expression in line with previous studies [35,59]. In addition, NKG2DL expression was not modulated by GS-9620 that, to our knowledge, has never been tested for this activity. When PRO was combined, the capacity of VOR to up-modulate ULBP2 was further increased, whereas ULBP2 expression was not higher than with HDACi alone in the ROM/PRO and PAN/PRO combinations (Figure 1E). Adding PRO had distinct effects on HDACi-induced MICA/B up-modulation since this was partially reduced in VOR/PRO and ROM/PRO cultures but slightly increased in the PAN/PRO combination. Moreover, by including BOR in HDACi-treated cultures, we observed an increment of MICA/B and ULBP2 up-modulation induced by any HDACi, although not statistically significant for VOR/BOR. Finally, the inclusion of GS-9620 in HDACi-stimulated cultures resulted in a drastic reduction of NKG2DLs up-modulation (Figure 1E).

Overall, in the J1.1 cell system, VOR and ROM performed much better than PAN and similarly to PRO at reactivating latent HIV, whereas BOR and GS-9620 were ineffective; simultaneously, VOR and ROM induced expression of both MICA/B and ULBP2 to a higher extent as compared with PAN, while PRO and BOR only up-regulated ULBP2 and GS-9620 had no effect on either NKG2DL. Moreover, association of an HDACi with a distinct LRA showed that combination of VOR and ROM with either PRO or BOR had the best potential in terms of efficient HIV reactivation and NKG2DL up-modulation. Lastly, combining GS-9620 with VOR, ROM, or PAN unexpectedly impaired the capacity of each HDACi to induce latent HIV as well as NKG2DL expression, hence GS-9620 was excluded from further analysis.

### 2.2. Viability of T and NK Cells within PBMCs Exposed to Single HDACi or BOR

**Figure 2:**
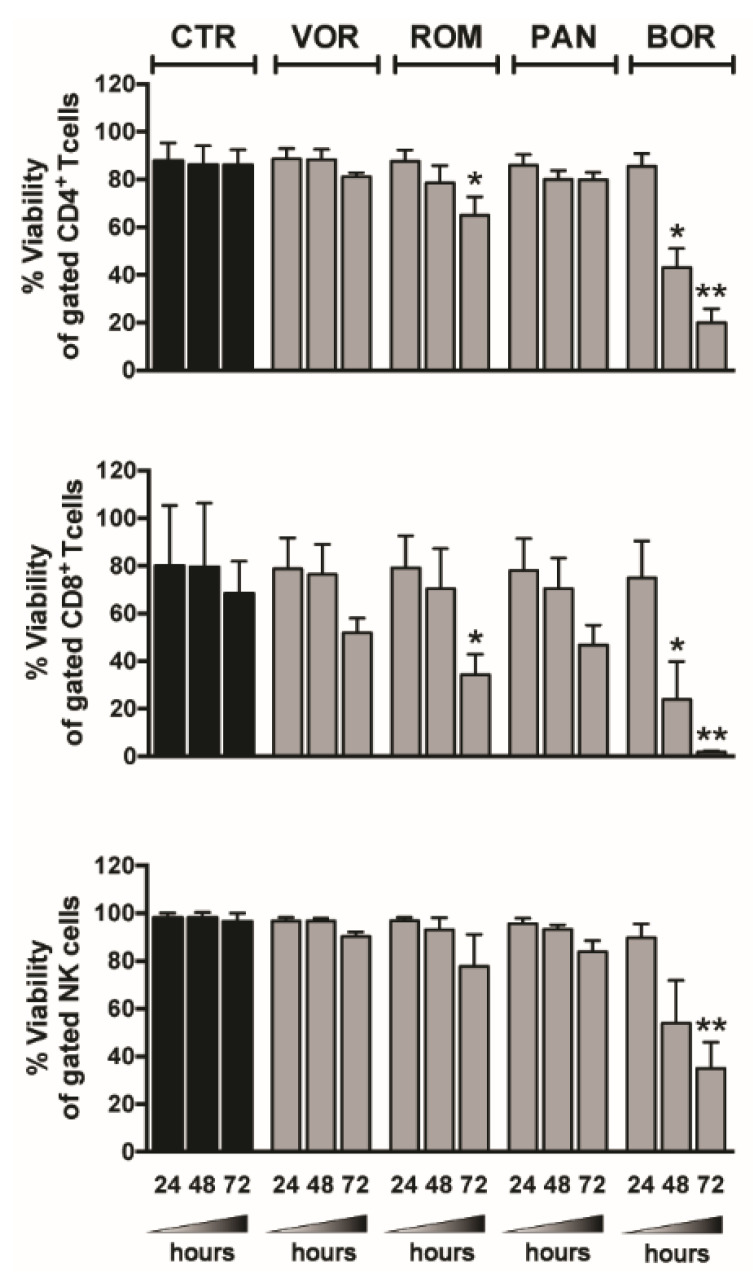
Lymphocyte viability in LRA-treated PBMCs. PBMCs were cultured in medium alone (control, CTR) or supplemented with 334 nM VOR, 10 nM ROM, 20 nM PAN, 5 nM BOR; after 24, 48, and 72 h, the viability of CD4^+^ T (top), CD8^+^ T (center), and NK cells (bottom) was examined by LIVE/DEAD staining and flow cytometry analysis of gated CD3^+^CD4^+^, CD3^+^CD8^+^, and CD3^−^CD56^+^CD16^+/−^ cells, respectively. Bars represent mean ± SEM (*n* = 4). * *p* < 0.05, ** *p* < 0.01 by paired *t*-test.

We previously showed that 1 µM PRO had no toxic effects on T or NK cells [35], as also reported by other groups [29,31], but concerns on the potential toxicity of HDACi and BOR on T or NK cells were raised in some studies [27,28,31,60,61]. Then, we cultivated PBMCs of healthy donors in medium supplemented with clinically relevant doses of VOR (334 nM), ROM (10 nM), PAN (20 nM), and BOR (5 nM) [27,62] and measured overtime (24, 48, and 72 h) the frequency of live cells within gated CD4^+^ T, CD8^+^ T, and NK cells (Figure 2). We found that viability of CD4^+^ T and NK cells was slightly affected by VOR and PAN (reduced by 6–13%) and modestly decreased by ROM (~20% reduction) compared with untreated cultures at 72 h, whereas by that time all HDACis showed toxicity in CD8^+^ T cells, with ROM having the strongest effect (50% reduction). When PBMCs were exposed to BOR, the viability of T and NK cells was strongly affected starting at 48 h and further decreased by 75–65% for CD4^+^ T and NK cells and 97% for CD8^+^ T after 72 h.

### 2.3. PRO Allows for Survival of HDACi-Reactivated HIV^+^ T Cells and Has Additive Effects on NKG2DL Expression

**Figure 3:**
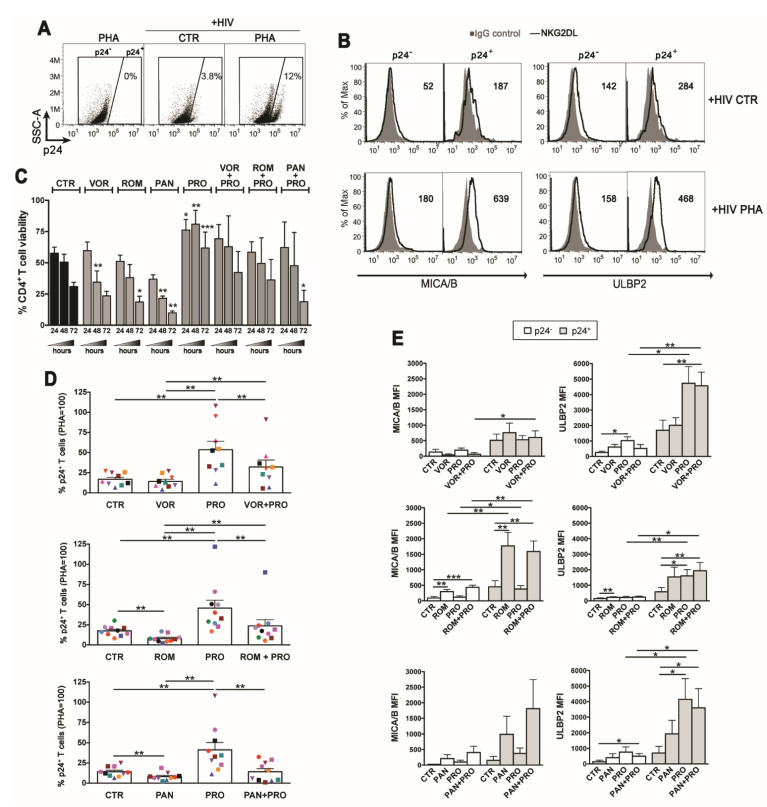
Impact of the HDACi/PRO combinations in viability, viral reactivation, and NKG2DLs expression in latently HIV-infected CD4^+^ T cells. HIV latency was established in CD4^+^ T cells freshly isolated from healthy donors (see Materials and Methods), then cells were exposed to LRAs for 3 days prior 2-color flow cytometry analysis of cell-surface MICA/B and ULBP2 expression in p24^-^ and p24^+^ cells. (**A**) Representative dot plots show the frequency of p24^+^ cells in control (CTR) and PHA-stimulated cultures gated by setting non-infected PHA-stimulated cells at 0%. (**B**) Histograms show MICA/B and ULBP2 fluorescence (solid line) in gated p24^-^ and p24^+^ cell populations measured in representative control and PHA-stimulated cell samples. Signals obtained with control IgG (filled histograms) and the ligand-specific MFI value, subtracted of the MFI value of isotype control, are shown. (**C**–**E**) Cells of several donors were cultivated in absence of stimuli (CTR) or with VOR (334 nM), ROM (10 nM), PAN (20 nM), PRO (1 µM), or combinations of each HDACi with PRO. (**C**) T cell viability (mean ± SEM, *n* = 3) was examined at 24, 48, and 72 h by LIVE/DEAD staining. (**D**) The frequency of p24^+^ cells harboring reactivated virus was evaluated for all conditions in 9–10 independent experiments (each donor is represented with distinct symbol) and expressed relatively to PHA set at 100%. (**E**) The cell-surface MICA/B and ULBP2 expression was analyzed on p24^-^ and p24^+^ cells as described in (**B**) in 5–8 independent experiments. Bars represent mean ± SEM. Statistics was performed using paired *t*-test (**C** and **E** panels) and Wilcoxon test (**D** panel) for parametric and non-parametric distributions, respectively. * *p* < 0.05, ** *p* < 0.01, *** *p* < 0.001.

Based on the results above on BOR toxicity, we decided to assess in latently HIV-infected CD4^+^ T cells HDACi combinations with PRO but not with BOR. Using a previously described method [40], latent HIV infection was established in primary resting CD4^+^ T cells derived from healthy donors and pre-treated with CCL19, then cells were cultivated for 72 h in the presence of HDACi, PRO, HDACi/PRO combinations, 10 μg/mL of PHA (for maximal stimulation), or DMSO vehicle as control, prior analysis by flow cytometry of HIV reactivation and cell-surface MICA/B and ULBP2 expression on cells with (p24^+^) and without (p24^−^) reactivated virus (a representative analysis is shown in Figure 3A,B). To optimize detection on primary T cells of MIC proteins, provided their elevated genetic polymorphism and inter-individual variability, T cells were simultaneously labeled with two mAbs that efficiently recognize MICA (AMO1) and MICB (MAB1599) molecules. As shown in a representative analysis comparing control with PHA-stimulated cultures (Figure 3B), expression of MICA/B and ULBP2 was higher on p24^+^ cells if compared with p24^-^ cells, in line with the capacity of HIV to up-regulate NKG2DLs [42,43,44,45,46]. Initially, we monitored overtime the viability of latently infected T cells, showing that it progressively diminished in unstimulated cultures, was further reduced in cultures treated with VOR and, particularly, with ROM and PAN (25%, 40%, and 70% reduction, respectively, compared with control at 72 h), while it was preserved in cultures supplemented with PRO (2-fold higher than in control at 72 h, Figure 3C). Importantly, in cultures with HDACi/PRO combinations, the viability of T cells was maintained at levels comparable to control cultures with the exception of PAN/PRO which was significantly reduced compared to control, indicating that the pro-survival activity of PRO contrasted the toxic effect of VOR and ROM but not that of PAN. As expected, we found that PRO, although less efficiently than PHA, induced the appearance of p24^+^ cells above the levels of spontaneous HIV reactivation (50 ± 7% vs. 17 ± 2% p24^+^ cells in control cultures, considering 100% the frequency of p24^+^ in PHA-treated cultures, Figure 3D). On the other hand, as compared to control cultures, the frequency of p24^+^ cells in cultures treated with single HDACi was similar for VOR (14 ± 3%) and significantly reduced for ROM and PAN (both 8% ± 1%; Figure 3D), which was likely due to the toxicity of these compounds (Figure 3C). When HDACis were used in combination with PRO, hence preserving T cell viability, HIV reactivation was higher than in single HDACi-treated cultures or in untreated cultures, although only VOR/PRO vs. VOR (32 ± 9% vs. 8% ± 1%) and ROM/PRO vs. ROM (24 ± 8% vs. 8% ± 1%) reached statistical significance; however, each HDACi/PRO combination reactivated HIV less efficiently than PRO alone (Figure 3D). Therefore, the simultaneous stimulation with PRO allowed for survival of p24^+^ cells treated with VOR or ROM but not PAN, yet combination with any HDCAi reduced the frequency of p24^+^ cells reactivated by PRO.

As to NKG2DLs, their expression in stimulated T cell cultures was enhanced in a drug-dependent manner and to a donor-to-donor variable extent. By testing several donors, we found that HDACis induced an overall MICA/B and ULBP2 up-modulation on p24^+^ cells, whereas the effect on p24^-^ cells was negligible (Figure 3E); above all, a strong and statistically significant increase of MICA/B expression was induced by ROM. Besides, stimulation with PRO had no effect on MICA/B but greatly enhanced ULBP2 expression on p24^+^ cells. Finally, in HDACi/PRO combinations, NKG2DLs up-modulation on p24^+^ cells were mostly maintained at the highest level induced by single treatments without showing major cooperative or antagonistic effects between drugs. In summary, which NKG2DL was induced in T cells that exit viral latency and the extent of ligand up-modulation depended on the nature of the drug, with MICA/B and ULBP2 being significantly up-modulated only by ROM and by PRO, respectively.

### 2.4. LRAs Effects on NK Cells

**Figure 4:**
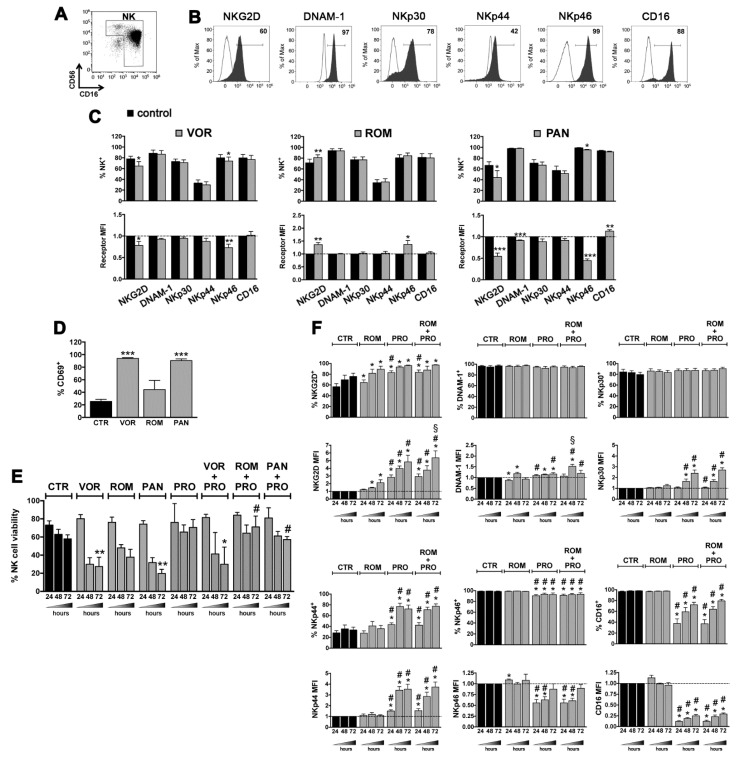
LRAs effects on NK-cell viability and phenotype. (**A**–**D**) NK cells, which include CD56^bright^CD16^+/−^ and CD56^dim^CD16^+^ cells, were isolated by negative selection from PBMCs of healthy donors reaching ~95% purity as shown in a representative flow cytometry analysis (**A**). Purified NK cells were cultured in medium alone (control, CTR) or supplemented with 334 nM VOR, 10 nM ROM, 20 nM PAN, 5 nM BOR for 24 h, then cells were analyzed by flow cytometry to measure the expression of various NK-cell markers. (**B**) The percentage of NKG2D^+^, DNAM-1^+^, NKp30^+^, NKp44^+^, NKp46^+^, and CD16^+^ cells among control NK cells is shown (filled gray histograms) together with control IgG signal (open histograms) for a representative experiment. (**C**) For each receptor, the frequency of positive cells and MFI (relatively to control MFI set to 1) was measured and mean ± SEM of at least 4 independent experiments is shown. (**D**) After 24 h of drug exposure, the percentage of CD69^+^ NK cells was evaluated in 4 donors (mean ± SEM). (**E**) Purified NK cells were cultivated in the presence of VOR, ROM, PAN, or PRO (1 µM) and with combinations of each HDACi with PRO. The NK-cell viability was examined at 24, 48, and 72 h by LIVE/DEAD staining. Bars represent mean ± SEM obtained from at least 3 independent donors. (**F**) Purified NK cells were treated with ROM and PRO, either alone or in combination, and analyzed after 24, 48, and 72 h for the expression of NK-cell receptors as described in panel (**C**). Bars show mean ± SEM (*n* = 6). Statistics was performed using paired Wilcoxon and *t*-test for non parametric and parametric distributions, respectively. Versus control (CTR): * *p* < 0.05; ** *p* < 0.01; *** *p* < 0.001. Versus ROM alone: ^#^ *p* < 0.05. Versus PRO alone: ^§^ *p* < 0.05.

To allow NK cell-mediated elimination of T cells harboring reactivated HIV, it is important to employ LARs devoid of negative effects on the repertoire of activating NK cell receptors. In a previous study, we analyzed NK cells purified from healthy donors and cultivated 20 h with PRO for the expression of activating receptors, including NKG2D, DNAM-1, NKp30, NKp44, NKp46, and CD16, and of the CD69 early activation marker, showing that PRO induced up-modulation of NKG2D, NKp44, and CD69 and loss of CD16, hence a phenotype typically associated with strong NK-cell activation [35]. Here the same analysis was performed using VOR, ROM, or PAN that, in previous studies, showed incongruous effects on NK cells [30,31,32,33]. We found that VOR and PAN induced a significant down-regulation on NK cells of NKG2D and NKp46 and strongly increased the frequency of CD69^+^ cells, while ROM had the opposite effect of up-regulating both NKG2D and NKp46 without a significant increase of CD69 expression (Figure 4A–D). Next, we investigated the impact of combinations of HDACi with PRO on purified NK cells by first analyzing their viability at 24, 48, and 72 h post-exposure. Of note, the toxicity of single HDACi on NK cells was more evident in purified NK-cell cultures (Figure 4E) as compared with PBMCs cultures (Figure 2), especially for VOR and PAN that killed between 50% and 75% of cells at 48–72 h; most importantly, the addition of PRO, which by itself preserved NK-cell viability, resulted in the full recovery of the viability of NK cells simultaneously exposed to ROM or PAN, an effect that was not observed with the VOR/PRO combination (Figure 4E). Then, considering that ROM but not PAN was devoid of negative effects on NK cell receptors, we examined overtime the phenotype of NK cells exposed to the ROM/PRO combination. Results showed that NKG2D expression was increased with the 2-drug combination by about 5-fold at 72 h, an induction significantly higher as compared with single drug treatments (Figure 4F). Moreover, PRO-induced up-modulation of NKp30 and NKp44 was maintained in the ROM/PRO combination and a trend towards increased expression of DNAM-1 (significantly higher DNAM-1 MFI at 48 h) was found comparing individual drugs with their combination. We also observed a negative effect of PRO on NKp46 and CD16 expression that was retained also when ROM was present; however, these inhibitory effects of PRO were progressively attenuated overtime, with the expression level of NKp46 and CD16 being fully and partially recovered at 72 h, respectively (Figure 4F).

### 2.5. Impact of the ROM/PRO Combination on NK-Cell Mediated Suppression of CD4^+^ T Cells That Exit from Viral Latency

**Figure 5:**
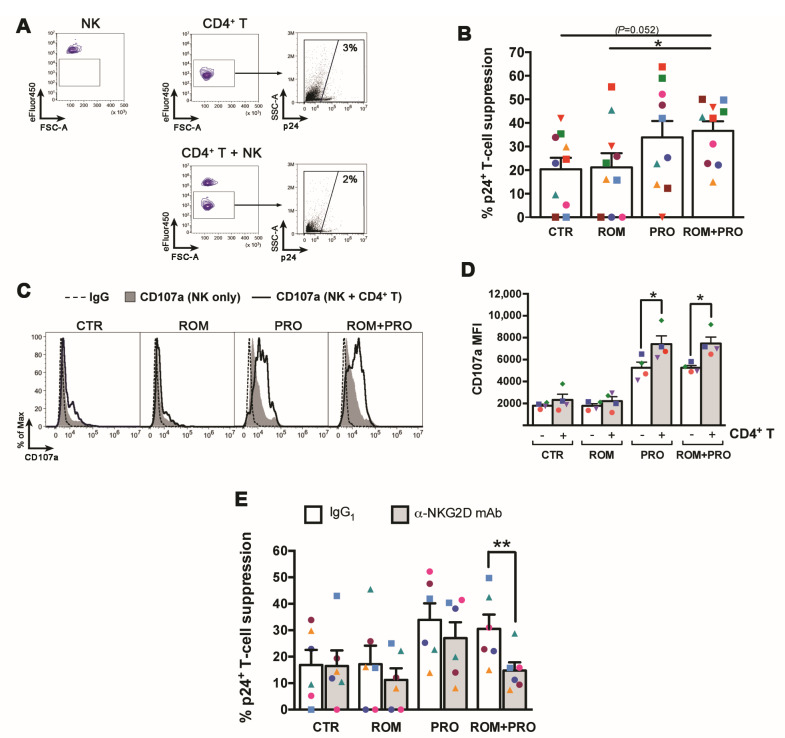
Clearance of reactivated HIV^+^ T cells by NK cells in the presence of Prostratin and/or Romidepsin. A viral suppression assay was set up in which latently infected CD4^+^ T cells (targets, T) were stimulated with ROM, PRO, ROM/PRO or not stimulated (CTR) for 72 h with and without the addition in the last 18 h of autologous eFluor450-labeled, equally stimulated/not stimulated NK cells (effectors, E) at an E:T ratio of 1:1. The T and T+NK cell cultures were analysed by flow cytometry to measure the frequency of p24^+^ cells among gated eFluor450^-^ targets and calculate the percentage of NK-cell mediated suppression (see Materials and Methods). (**A**) The gating strategy is shown for a representative set of not stimulated autologous NK cells and CD4^+^ T cells cultivated separately or together for the last 18 h. (**B**) The % of p24^+^ cell suppression by NK cells in CTR, ROM, PRO, and ROM/PRO co-cultures was calculated in 10 independent experiments. (**C**,**D**) NK cell degranulation was measured by including anti-CD107a mAb or IgG control to T+NK and NK cell cultures in the last 18 h; (**C**) CD107a expression among eFluor450^+^ NK cells cultivated without (filled gray) or with CD4^+^ T cells (open black histograms) is shown together with control IgG signal (dashed line) for a representative experiment; (**D**) CD107a MFI measured in 4 independent experiments is shown. (**E**) The suppression of p24^+^ cells by NK cells pre-incubated with anti-NKG2D mAb or control IgG_1_ was measured in 6 different donors as described in panels (**A**,**B**). Bars represent mean ± SEM. Each symbol corresponds to one donor. * *p* < 0.05, ** *p* < 0.01 by paired *t* test.

Overall, our results suggest that ROM/PRO combined treatment may favor NK cell-mediated clearance of latently infected T cells by its capacity to induce expression of both MICA/B and ULBP2 on T cells that exit from latency and of the NKG2D receptor on NK cells. To verify this hypothesis, we set up a viral suppression assay in which latently infected CD4^+^ T cells were stimulated with ROM and/or PRO or not stimulated for 48 h, then transferred on a pellet of autologous NK cells that had been exposed separately to the same 48 h-treatment and further cultivated for 18 h before analysis of p24^+^ cell frequency in gated T cells (see Materials and Methods and gating strategy in Figure 5A). Data obtained with 10 donors showed that co-culture with autologous NK cells resulted in a median 20 ± 5% suppression of spontaneously reactivated p24^+^ cells in the absence of treatments; analogous NK cell-mediated suppression levels were found in ROM co-cultures (21 ± 6%), with only 3/10 donors showing increased clearance of p24^+^ cells, 6/10 donors showing reduced killing, and 1/10 donors being unresponsive (Figure 5B). On the other hand, NK-cell mediated viral suppression was 34 ± 7% in the presence of PRO (increased in 8/10 donors) and 37 ± 4% with ROM/PRO combination (increased in 7/10 donors), this latter being higher than in ROM-treated and untreated co-cultures in a statistically significant and nearly significant (*p* = 0.052) manner, respectively (Figure 5B). Therefore, notwithstanding interindividual variation in the response to treatments, simultaneous exposure of latently infected T cells and NK cells to ROM did not stimulate NK-cell mediated suppression of T cells that exit viral latency, whereas PRO and, even better, ROM/PRO exposure resulted in increased suppression. To confirm that the reduction of 24^+^ cells reflected NK-cell cytotoxic activity, expression of the CD107a degranulation marker was analyzed in NK cells following the 18 h culture in various conditions (Figure 5C). Results showed that the presence of CD4^+^ T cell targets significantly incremented CD170a levels in PRO and ROM/PRO but not in CTR and ROM cultures (Figure 5C,D), therefore p24^+^ cell suppression could be confidently associated with NK cell-mediated killing in this assay. Finally, to assess the contribution of the NKG2D/NKG2DL axis in this HIV suppression assay, 6 donors were tested by pre-treating NK cells with anti-NKG2D blocking mAb or isotype IgG control prior addition to T cell cultures. Figure 5E shows that blocking NKG2D significantly reduced the capacity of NK cells to suppress HIV^+^ reactivated cells in ROM/PRO-stimulated cultures (48% reduction), had a lower effect in cultures exposed to ROM or PRO (35% and 20% reduction, respectively) and no effect in control cultures, indicating that ROM/PRO enhanced NKG2D-mediated killing of p24^+^ targets by NK cells as compared to single drug treatments.

## 3. Discussion

Initial HIV-1 eradication trials in ART patients have failed to significantly reduce the size of the latent viral reservoir and clearly demonstrated the need for interventions improving both latency reversal and killing of reactivated HIV^+^ cells through host immune responses. In the present study we searched for 2-LRA combinations, including one HDACi and one functionally distinct LRA that could enhance NK-cell mediated clearance of reactivated HIV^+^ T cells via the potentiation of the NKG2D/NKG2DL axis. The rationale behind this approach stands on the reported ability of HDACis and different candidate LRAs to induce both expression of latent HIV-1 and NKG2DLs, thus potentially enhancing the virus-mediated NKG2DL induction and increasing the susceptibility of T cells that exit viral latency to recognition and killing by NK cells via their activating NKG2D receptor.

We focused on three HDACis, VOR, ROM, and PAN, that have been already administered to HIV-infected patients and vary considerably in their biochemical properties, specificity, and efficacy. ROM is generally classified as specific for class I HDACs, which are considered the most important in controlling integrated HIV transcription [63], as opposed to the VOR and PAN pan-HDACis that target class I, II, and IV HDACs [64]. However, in a comparative examination against recombinant HDAC isoenzymes, ROM demonstrated the strongest activity against HDACs belonging to I, IIb, and IV classes [56], hence it is also referred to as pan-HDACi in some studies [65]. As compared to ROM, VOR and PAN displayed a weaker activity not only with class I HDACs, possibly accounting for their lower latency reversal activity in primary T cell models, but also against the majority of HDAC enzymes, with the exception of the class IIb HDAC6 enzyme, with PAN being the stronger inhibitor [56].

Herein, using the J1.1 latently HIV-infected T cell line, we initially checked association of VOR, ROM, or PAN, with either PRO, BOR, or GS-9620, showing that VOR and ROM combined with PRO or BOR had the best potential in terms of HIV reactivation, thus confirming and extending previous studies [20,21,22,23,24,25,26,66], and of MICA/B and/or ULBP2 induction. On the other hand, PAN, which by itself had a modest activity on HIV, when combined with PRO resulted in no major changes in NKG2DL expression and in a strong reduction of HIV reactivation induced by PRO alone. Interestingly, this result is in line with a previous study showing that PAN antagonized a distinct PKCa, Ingenol, at reactivating HIV ex vivo in CD4^+^ T cells [67]. This antagonism could be due to the superior capacity of PAN to inhibit the deacetylase of HSP90, HDAC6, as compared to VOR or ROM [56], which leads to dissociation of the HSP90/IKK complex required for NF-κB activation by PKCa [68,69]. Another remarkable result of the 2-drug screening in J1.1 cells consisted of the strong antagonistic effect of GS-9620 on both HIV reactivation and NKG2DL induction activities of HDACis; actually, it has been demonstrated that GS-9620 reverses HIV latency in CD4^+^ T cells in a paracrine manner through the effect of cytokines [52,53], yet a direct effect in T cells that exit from viral latency was not investigated. The antagonistic effect of GS-9620 on HDACi-induced HIV reactivation found herein might be explained by the fact that TLR-7 engagement in CD4^+^ T cells impedes activation of Jun, a component of the AP-1 transcription factor that enhances HIV RNA elongation in CD3/CD28-reactivated latently infected T cells [70,71]. Analogously, since AP-1 was shown to positively stimulate *NKG2DL* transcription [72], it is plausible that inhibition of AP-1 by GS-9620 could interfere with HDACi-induced ligand expression. Despite the mechanism is presently undefined, the strong antagonistic effects of GS-9620 discouraged further testing in this study.

To extend our analysis to primary cells, we first measured the viability of PBMCs from HIV-negative donors cultivated with single LRAs, finding a dramatic toxicity of BOR in CD4^+^ T cells as well as in CD8^+^ T and NK cells starting at 48 h of treatment; deleterious effects of BOR on the viability of primary CD4^+^ T cells derived from healthy individuals or ART patients were also reported in recent studies [25,73], therefore we decided to exclude BOR from further analysis. Moreover, we found that viability of CD4^+^ T and NK cells was minimally, if not at all, affected in PBMC cultures exposed to individual HDACi for up to 72 h, yet it was significantly reduced when tested in purified CD4^+^ T cells latently infected in vitro or NK cell cultures. Of note, simultaneous addition of PRO, which by itself increased the viability of purified CD4^+^ T and NK cells, attenuated the toxic effects of VOR or ROM in CD4^+^ T cells as well as the toxicity of ROM and PAN in NK cells. Analogously, a recent work showed that PRO and the TPPB and (-)-Indolactam V PKCas reduced the toxicity of various HDACis (i.e., Belinostat, Givinostat, AR-42, PCI-24781) when combined in PBMC cultures [26]. Our results suggest that activation of the PKC signaling pathway may overcome HDACi-induced intrinsic apoptotic pathways depending on the cell type and on the nature of the HDACi used. Further work is necessary to elucidate these finding and extend the analysis to CD8^+^ T cells whose viability was strongly affected by HDACi in this as in previous studies [27,28,60].

By measuring appearance of p24^+^ cells in cultures of CD4^+^ T cells that were latently infected then exposed to LRAs for 72 h, we found that PRO efficiently reactivated HIV, which is in line with PKCas being the strongest LRAs in ex vivo or in vitro assays [18,22], whereas VOR was ineffective and ROM and PAN even reduced the level of spontaneous viral reactivation. We interpreted the inefficacy of HDACis at reverting HIV latency as a consequence of their intrinsic toxicity under the tested experimental conditions. Indeed, the addition of PRO to HDACi, which preserved the viability of cells exposed to VOR or ROM but not PAN, resulted in higher frequency of p24^+^ cells with the exclusion of the PAN/PRO combination. However, all HDACi/PRO combinations reactivated HIV less efficiently than PRO alone, suggesting that HDACis partially antagonized the activity of PRO.

These results contrast with some former studies, though discrepancies may be explained by major methodological differences. In fact, various studies showing that VOR, ROM, or PAN reactivated HIV in ex vivo assays or in primary CD4^+^ T cell models of latency employed experimental conditions that preserved cell viability, such as ectopic expression of the anti-apoptotic factor Bcl2 [18,74], addition of anti-apoptotic agents [67], differentiation of memory T cell subsets [56], or stimulation with allogeneic cells [75], while we investigated HIV reactivation in the absence of any pro-survival factor or stimuli other than LRAs. When tested for latency reversal in ex vivo assays devoid of stimulatory factors, HDACis were ineffective or showed minimal activity as compared with PKCas or control PMA/Iono, especially in terms of mature HIV mRNA released in the culture supernatant [18,19,20,22,56,76]. In addition, one study demonstrated that ART patients’ CD4^+^ T cells super-infected in vitro and exposed to HDACis (i.e., ROM and Nanatinostat) did not induce degranulation of autologous HIV-specific CD8^+^ T cells [19], which is in agreement with the inability of HDACis to stimulate expression of the viral p24 antigen in our in vitro assay. On the other hand, HDACis were shown to synergize with PKCas, specifically ROM, VOR or valproic acid with PRO or BRY, at inducing the release of HIV mRNA by a fraction of T cell cultures derived from ART patients [20,22]. This synergism, which is at odd with our data, has been lately contrasted by a report demonstrating that VOR, ROM, or PAN, as opposed to bona fide class-I-specific HDACis, disrupted the latency reversing activity of PKCas in a primary T cell model, also providing evidence that VOR inactivated HSP90 and inhibited BRY-mediated NF-κB activation [65].

The response of LRA-exposed latently infected CD4^+^ T cells in terms of NKG2DL expression varied considerably between donors, yet overall showed that HDACis and PRO up-modulated at least one ligand (MICA/B and/or ULBP2) on reactivated p24^+^ cells as compared with p24^-^ cells or with p24^+^ cells spontaneously occurring in control unstimulated cultures, extending our previous work demonstrating that VOR and PRO cooperate with reactivated HIV at inducing ULBP2 expression [35,38]. Specifically, a significant up-regulation of MICA/B was induced by ROM, whereas PRO was ineffective on MICA/B but efficiently up-regulated ULBP2. In HDACi/PRO combinations, NKG2DL up-modulation on p24^+^ T cells was mostly maintained at the highest level induced by single drug treatments, suggesting that the effects of these distinct drugs on ligand expression were not cumulative.

In a ‘shock-and-kill’ scenario, NKG2DL expression exposes HIV^+^ T cells reactivated by LRAs to the recognition and killing by NK cells, given that their NKG2D-mediated responses are preserved by the administered drugs. Here we showed that VOR and PAN had deleterious effects in 24 h cultures of purified NK cells consisting in ample cell death, NKG2D and NKp46 down-regulation, and overall cell activation as measured by CD69 expression; conversely, ROM modestly reduced NK cell viability and up-regulated NKG2D and NKp46 receptors without inducing CD69. These results are only marginally consistent with previous studies describing a negative effect of PAN on all tested NK-cell receptors except NKp44, either NKp44 down-regulation or no effects for VOR and ROM, and a significant increase of %CD69^+^ cells by the three HDACis [31,32]. The effect of ROM was further analyzed overtime with the addition of PRO that alone induced strong NK cell activation by up-modulating NKG2D, NKp30, NKp44, and CD69, and by CD16 shedding [35]. The ROM/PRO combination resulted after 72 h in fully preserved cell viability (otherwise partially reduced by individual ROM treatment) and further increased NKG2D expression as compared with single drug treatments.

Results presented herein indicated that ROM/PRO combined treatment may enhance NK cell-mediated clearance of latently infected T cells by its capacity to induce expression of both MICA/B and ULBP2 on CD4^+^ T cells that exit from latency and of the NKG2D receptor on NK cells. To test this hypothesis, we set up a killing assay in which both targets (in vitro latently infected CD4^+^ T cells) and effectors (autologous NK cells) were exposed to ROM and/or PRO or untreated for the same period of time and in the absence of additional stimuli, in this respect mimicking the conditions that may occur in clinical settings. Importantly, this assay measured the capacity of NK cells to clear T cells that effectively emerged from latency in the last 24 h of 3-days exposure to LRAs as determined by viral proteins expression (p24^+^ cells). Of note, the ~20% NK-cell mediated clearance of reactivated HIV^+^ cells measured in untreated cultures in 10 donors was overall not changed in parallel ROM cultures though, more specifically, virus suppression was increased by ROM in 3 donors, reduced in 6 donors and not affected in 1 donor. These results indicated that stimulation of the NKG2D/NKG2DLs axis could be contrasted by some deleterious effect(s) of ROM, such as inhibition of NK cell cytotoxicity described earlier [31,32,35], with a net balance between these opposite activities that varied from one donor to another. However, when ROM was combined with PRO, more donors responded positively, with an overall significantly higher NK cell-mediated suppression of reactivated p24^+^ cells. Although not reaching statistical significance, the sole treatment with PRO resulted in enhanced virus suppression, in agreement with previous reports in which NK cells were exposed to PRO only before or during the killing assay [31,32,35]. Importantly, by pre-incubating NK cells with anti-NKG2D blocking antibody, we were able to demonstrate that ROM/PRO augmented the function of NKG2D in NK cell-mediated suppression of p24^+^ target cells as compared with single drug treatments, thus providing evidence that the pathways mediating NK cell recognition and killing of T cells harboring reactivated HIV can be specifically modulated by the administered LRAs.

The present study has limitations since experiments have been performed using in vitro models of HIV latency, hence further investigations in ex vivo ART patient-derived cells are needed. At any rate, in vitro experiments have been so far instrumental to address various important aspects of latency reversal and there is an overall good concordance between studies based on experimental models of HIV latency in primary CD4^+^ T cells and ex vivo studies when the same conditions were adopted.

Overall, our results endorse the importance of studying the impact of LRAs on the capacity of NK cells to kill CD4^+^ T cells that exit from HIV latency and provide a path for NK cell potentiation towards the clearance of the viral reservoir. On one hand, our data add novel evidence that single HDACi administration is unlikely to be effective in a ‘shock-and-kill’ strategy on the basis of their general toxicity in primary CD4^+^ T and NK cells as well as their inability to elicit NK cell-mediated clearance of reactivated HIV^+^ T cells when both cell types are exposed to the moderately toxic ROM. Apparently, the capacity of ROM to enhance expression of MICA/B on p24^+^ targets and NKG2D on NK cells was not sufficient to induce killing. On the other hand, as shown for PRO combined with ROM, co-administration of a PKCa has the potential to negate the inhibitory effect of HDACi on cell viability and enhance HIV reactivation, as well as to up-modulate ULBP2 and its cognate NKG2D receptor, ultimately boosting the NKG2D-mediated viral suppression by NK cells. On the basis of preliminary tests in J1.1 cells and/or PBMCs, the proteasome inhibitor BOR and the GS-9620 TLR-7 agonist were excluded from further analysis in combination with HDACi because of their toxicity and antagonistic effects on HIV reactivation/NKG2DL expression, respectively.

At present, HDACis and other drugs that entered as LRAs into clinical trials were selected on the basis of their known safety in the treatment of cancer or different diseases. However, novel LRAs with improved efficacy, specificity, and safety profiles are continuously being developed, offering an enormous potential for optimizing strategies towards an HIV cure. Among candidate LRAs there are various synthetically produced analogs of PRO that are more accessible than the original naturally-occurring compound and, importantly, are more efficacious as LRAs in ex vivo assays [77]. Analogously, several synthetic BRY analogs (bryologs) potentiated in their capacity to reactivate HIV in vitro and ex vivo and better tolerated in humanized mouse models have been developed [78]. In addition, a plethora of compounds derived from the ingenol ester PKCa showing tolerable levels of toxicity and significantly improved efficacy as LRAs in CD4^+^ T cells from ART patients were made available [79]. Very promising LRAs also include class I-specific HDACis such as Entinostat, which was shown to selectively inhibit HDAC-1 and -2 while preserving the activity of HDACi isoenzymes necessary for maximal HIV reactivation and without disrupting the PKCa activity [65]. Interestingly, Entinostat enhanced NK cell-mediated killing of tumors through both induction of MICA/B on target cells and NKG2D up-modulation on NK cells in a cancer study [34], hence the impact of Entinostat on the NKG2D/NKG2DL pathway in the context or NK cell clearance of reactivated HIV is worth being investigated in future work. Of note, evidence was recently provided that ingenol derivatives and bryologs can synergize with HDACis, including Entinostat, at reversing HIV latency in ex vivo or in vitro assays [80,81].

In conclusion, while the present study describes that HIV latency reversal and NK-cell mediated clearance of T cells harboring reactivated virus could be simultaneous addressed by a targeted HDACi/PKCa combination, this strategy should be optimized in further studies using more robust and less toxic recently identified compounds that offer great promise for virus eradication in HIV-infected people.

## 4. Materials and Methods

### 4.1. Cells, Antibodies, and Reagents

J1.1 cells (NIH AIDS Reagent Program) and primary cells were maintained in complete RPMI 1640 medium supplemented with 10% fetal bovine serum, 0.2 mM L-glutamine, and 100 units/mL penicillin-streptomycin (all from Euroclone, Pero, Italy). PBMCs were obtained by Ficoll separation of buffy coats from a donor bank. Primary NK and CD4^+^ T cells were isolated from PBMCs by negative selection with cell-type specific EasySep CD4^+^ T-cell Enrichment Kit (Stem Cell Technologies, Vancouver, Canada) according to manufacturer’s protocol. The purity (~95%) of isolated NK (CD3^-^CD56^+^CD16^-/+^) and CD4^+^ T cells (CD3^+^CD4^+^) was assessed by immunolabeling and FACS analysis.

For flow cytometry, isotype control IgG (BD Pharmingen, San Diego, CA, USA) and the following mouse monoclonal antibodies (mAbs) were used: CD3/AlexaFluor700 (UCHT1), CD56/PerCpCy5.5 (B159), CD16/BV510 (3G8), from BD Pharmingen; NKG2D(CD314)/PE (1D11), CD16/APC-eFluor780 (CB16), CD107a/FITC (H4A3) from eBioscience (San Diego, CA, USA); CD8/APC (HIT8a), CD69/PE (FN50), DNAM-1(CD226)/FITC (11A8), NKp30/APC, (P30-15), NKp44/PE (P44-8), NKp46/PE-Cy7 (9E2), NKG2D/Bv785 (1D11), MICAB/APC (6DA), from BioLegend (San Diego, CA, USA); p24/FITC (KC57) from Beckman Coulter (Brea, CA, USA); CD4/PE (MT310) from DAKO (CA, USA); MICA (AMO1) from BamOmaB (Gräfelfing, Germany); MICB (MAB1599), ULBP2/5/6 (MAB1298) and ULBP2-5-6/PE (165903) from R&D Systems (Minneapolis, MN, USA). As a secondary antibody, Alexa647- or Alexa488-coniugated goat anti-mouse IgG (GAM) (Invitrogen/Thermo Fisher Scientific, Waltham, MA, USA) was used.

The anti-NKG2D (149810; R&D Systems) mAb or isotype control IgG_1_ (Eurobiosciences, Friesoythe, Germany) was used as blocking antibody in NK-cell killing assay.

Where indicated, cells were treated with 10 µM or 335 nM suberoylanilide hydroxamic acid (Vorinostat, VOR), 20 or 10 nM Romidepsin (ROM), 20 nM Panobinostat (PAN), 1 µM Prostratin (PRO), 3 µM GS-9620 (GS), 5 nM Bortezomib (BOR), 100 ng/mL phorbol-12-myristate-13-acetate (PMA), 1 µg/mL Ionomycin (IONO), 10 µg/mL phytohemagglutinin (PHA), or with equivalent amounts of dimethyl sulfoxide (DMSO) when used as solvent (all from Sigma-Aldrich, St. Louis, MO, USA). Other reagents used were: 29 nM CCL19 (R&D Systems), cell proliferation dye eFluor450 (Thermo Fisher Scientific), and Golgi stop (BD Pharmingen).

### 4.2. Flow Cytometry

To assess viability, cells were stained with LIVE/DEAD fixable NEAR-IR dead cell stain kit according to manufacturer’s protocol (Life Technologies/Thermo Fisher Scientific). The cell-surface and intracellular staining procedures were performed as described previously [38]. Immunolabeled cells resuspended in 1% paraformaldehyde (PFA) were acquired on Cytoflex (Beckman Coulter). Positive cell gating was set using fluorescence minus one control (FMO). Mean fluorescence intensity (MFI) was subtracted of the value obtained with isotype control antibody. Data analyses were performed using Kaluza v2.1 (Beckman Coulter) or FlowJo v10 (BD Pharmingen).

### 4.3. Establishment and Reactivation of Latently Infected CD4^+^ T Cells

Resting CD4^+^ T cells cultures latently infected with HIV-1 were established and then reactivated as previously described with minor modifications [40]. Briefly, purified CD4^+^ T cells cultivated with 29 nM CCL19 (R&D Systems) for 1–3 days, were infected by spinoculation with 300 ng p24/10^6^ cells of NL4-3 HIV-1 (NIH AIDS Reagent Program) pseudotyped with vesicular stomatitis virus glycoprotein (VSV-G), washed, and placed back in culture in complete medium alone. At day 3 post-infection, latently infected CD4^+^ T cells were exposed to an HDACi (335 nM VOR, 10 nM ROM, or 20 nM PAN), either alone or in combination with 1 µM PRO, to 10 μg/mL phytohemagglutinin (PHA), or non-stimulated. Finally, cells were harvested and analyzed by FACS for their viability (at 24, 48, and 72 h post-stimulation) and for the expression of cell-surface NKG2DLs and intracellular HIV p24 protein (72 h post-stimulation).

### 4.4. NK Cell-Mediated Killing of Reactivated HIV-1-Infected Cells

At day 3 post-infection, HIV-infected CD4^+^ T cells (targets, T) were stimulated with 10 nM ROM and/or 1 µM PRO or not treated (CTR); the same day, NK cells (effectors, E) were purified from an aliquot of cryopreserved PBMCs of the same donor, labeled with eFluor450 according to manufacturer’s protocol, and placed in culture with and without stimuli (ROM, PRO, ROM/PRO, CTR). At 48 h post-stimulation, an aliquot of target cell culture containing 2 × 10^5^ cells was transferred in a new well either empty (targets alone) or containing a pellet of 2 × 10^5^ autologous NK cells that have been cultivated separately for 48 h in the same treatment condition (E:T ratio of 1:1), gently mixed, and further cultivated for 18 h. Cells were then fixed/permeabilized and stained for p24. Finally, cells were acquired by FACS and the frequency of p24^+^ target cells (gated as eFluor450^-^) was analyzed. The percent NK cell-mediated suppression of p24^+^ cells was calculated with the following formula: 100 × [(%p24^+^ cells in targets − %p24^+^ cells in targets with effectors)/(%p24^+^ cells in targets)]. Where indicated, NK cells were incubated with anti-NKG2D blocking antibody or control IgG_1_ (1 µg/10^6^ cells) for 15 min prior co-culture with T cells. To measure NK-cell degranulation, anti-CD107a/FITC or isotype control IgG/FITC was added after the 48 h treatment to NK:T co-cultures and cultures of NK cells alone; after 1 h, also Golgi stop was added (1:1500 final dilution) and cells were further cultivated for 17 h prior analysis of CD107a expression in NK cells gated as eFluor450^+^.

### 4.5. Statistical Analysis

All experiments have been performed independently at least three times. GraphPad Prism v6.0 software (San Diego, CA, USA) was used to perform all statistical analyses. A value of *p* < 0.05 was considered statistically significant.

## Data Availability

The data that support the findings of this study are available from the corresponding author, [M.D.], upon reasonable request.

## Supplementary Materials

The following are available online at https://www.mdpi.com/article/10.3390/ijms22136654/s1.

## References

[1] Chun T.-W., Stuyver L., Mizell S.B., Ehler L.A., Mican J.A.M., Baseler M., Lloyd A., Nowak M.A., Fauci A.S. (1997). Presence of an inducible HIV-1 latent reservoir during highly active antiretroviral therapy. Proc. Natl. Acad. Sci. USA.

[2] Wong J.K., Hezareh M., Günthard H.F., Havlir D.V., Ignacio C.C., Spina C.A., Richman D.D. (1997). Recov-ery of Replication-Competent HIV Despite Prolonged Suppression of Plasma Viremia. Science.

[3] Finzi D., Blankson J.N., Siliciano J.D., Margolick J.B., Chadwick K., Pierson T.C., A Smith K., Lisziewicz J., Lori F., Flexner C. (1999). Latent infection of CD4+ T cells provides a mechanism for lifelong persistence of HIV-1, even in patients on effective combination therapy. Nat. Med..

[4] Kim Y., Anderson J.L., Lewin S.R. (2018). Getting the “Kill” into “Shock and Kill”: Strategies to Eliminate La-tent HIV. Cell Host Microbe.

[5] Spivak A.M., Planelles V. (2018). Novel Latency Reversal Agents for HIV-1 Cure. Annu. Rev. Med..

[6] Abner E., Jordan A. (2019). HIV “Shock and Kill” Therapy: In Need of Revision. Antiviral Res..

[7] Coiras M., López-Huertas M.R., Perez-Olmeda M., Alcami J. (2009). Understanding HIV-1 latency provides clues for the eradication of long-term reservoirs. Nat. Rev. Genet..

[8] Archin N.M., Liberty A.L., Kashuba A.D., Choudhary S.K., Kuruc J.D., Crooks A.M., Parker D.C., An-derson E.M., Kearney M.F., Strain M.C. (2012). Administration of Vorinostat Disrupts HIV-1 Latency in Patients on Antiretroviral Therapy. Nature.

[9] Elliott J.H., Wightman F., Solomon A., Ghneim K., Ahlers J., Cameron M.J., Smith M.Z., Spelman T., McMahon J., Velayudham P. (2014). Activation of HIV Transcription with Short-Course Vorinostat in HIV-Infected Patients on Suppressive Antiretroviral Therapy. PLoS Pathog..

[10] Archin N.M., Kirchherr J.L., Sung J.A., Clutton G., Sholtis K., Xu Y., Allard B., Stuelke E., Kashuba A.D., Kuruc J.D. (2017). Interval dosing with the HDAC inhibitor vorinostat effectively reverses HIV latency. J. Clin. Investig..

[11] Søgaard O.S., Graversen M.E., Leth S., Olesen R., Brinkmann C.R., Nissen S.K., Kjaer A.S., Schleimann M.H., Denton P.W., Hey-Cunningham W.J. (2015). The Depsipeptide Romidepsin Reverses HIV-1 Latency In Vivo. PLoS Pathog..

[12] Rasmussen T.A., Tolstrup M., Brinkmann C.R., Olesen R., Erikstrup C., Solomon A., Winckelmann A., Palmer S., Dinarello C., Buzon M. (2014). Panobinostat, a Histone Deacetylase Inhibitor, for Latent-Virus Reactivation in HIV-Infected Patients on Suppressive Antiretroviral Therapy: A Phase 1/2, Single Group, Clin-ical Trial. Lancet HIV..

[13] Spivak A.M., Andrade A., Eisele E., Hoh R., Bacchetti P., Bumpus N.N., Emad F., Buckheit R., McCance-Katz E.F., Lai J. (2014). A Pilot Study Assessing the Safety and Latency-Reversing Activity of Disulfi-ram in HIV-1-Infected Adults on Antiretroviral Therapy. Clin. Infect. Dis..

[14] Elliott J.H., McMahon J.H., Chang C.C., A Lee S., Hartogensis W., Bumpus N., Savic R., Roney J., Hoh R., Solomon A. (2015). Short-term administration of disulfiram for reversal of latent HIV infection: A phase 2 dose-escalation study. Lancet HIV.

[15] Gutiérrez C., Serrano-Villar S., Madrid-Elena N., Elias M.J.P., Martín M.E., Barbas C., Ruipérez J., Muñoz E., Muñoz-Fernández M., Ángeles C.T. (2016). Bryostatin-1 for latent virus reactivation in HIV-infected patients on antiretroviral therapy. AIDS.

[16] Vibholm L., Schleimann M.H., Højen J.F., Benfield T., Offersen R., Rasmussen K., Olesen R., Dige A., Agnholt J., Grau J. (2017). Short-Course Toll-Like Receptor 9 Agonist Treatment Impacts Innate Immunity and Plasma Viremia in Individuals With Human Immunodeficiency Virus Infection. Clin. Infect. Dis..

[17] Zerbato J.M., Purves H.V., Lewin S.R., Rasmussen T.A. (2019). Between a Shock and a Hard Place: Chal-lenges and Developments in HIV Latency Reversal. Curr. Opin. Virol..

[18] Bullen C.K., Laird G.M., Durand C.M., Siliciano J.D., Siliciano R.F. (2014). New Ex Vivo Approaches Dis-tinguish Effective and Ineffective Single Agents for Reversing HIV-1 Latency in Vivo. Nat. Med..

[19] Mota T.M., McCann C.D., Danesh A., Huang S.-H., Magat D.B., Ren Y., Leyre L., Bui T.D., Rohwetter T.M., Kovacs C.M. (2020). Integrated Assessment of Viral Transcription, Antigen Presentation, and CD8 + T Cell Function Reveals Multiple Limitations of Class I-Selective Histone Deacetylase Inhibitors during HIV-1 Latency Reversal. J. Virol..

[20] Reuse S., Calao M., Kabeya K., Guiguen A., Gatot J.S., Quivy V., Vanhulle C., Lamine A., Vaira D., Demonte D. (2009). Synergistic Activation of HIV-1 Expression by Deacetylase Inhibitors and Prostratin: Impli-cations for Treatment of Latent Infection. PLoS ONE.

[21] Burnett J.C., Lim K.I., Calafi A., Rossi J.J., Schaffer D.V., Arkin A.P. (2010). Combinatorial Latency Reacti-vation for HIV-1 Subtypes and Variants. J. Virol..

[22] Laird G.M., Bullen C.K., Rosenbloom D.I., Martin A.R., Hill A.L., Durand C.M., Siliciano J.D., Siliciano R.F. (2015). Ex vivo analysis identifies effective HIV-1 latency–reversing drug combinations. J. Clin. Investig..

[23] Pan X.Y., Zhao W., Wang C.Y., Lin J., Zeng X.Y., Ren R.X., Wang K., Xun T.R., Shai Y., Liu S.W. (2016). Heat Shock Protein 90 Facilitates Latent HIV Reactivation through Maintaining the Function of Positive Tran-scriptional Elongation Factor b (p-TEFb) Under Proteasome Inhibition. J. Biol. Chem..

[24] Albert B.J., Niu A., Ramani R., Marshall G.R., Wender P.A., Williams R.M., Ratner L., Barnes A.B., Kyei G.B. (2017). Combinations of isoform-targeted histone deacetylase inhibitors and bryostatin analogues display remarkable potency to activate latent HIV without global T-cell activation. Sci. Rep..

[25] Li Z., Wu J., Chavez L., Hoh R., Deeks S.G., Pillai S.K., Zhou Q. (2019). Reiterative Enrichment and Au-thentication of CRISPRi Targets (REACT) Identifies the Proteasome as a Key Contributor to HIV-1 Latency. PLoS Pathog..

[26] Curreli F., Ahmed S., Victor S.M.B., Debnath A.K. (2020). Identification of Combinations of Protein Kinase C Activators and Histone Deacetylase Inhibitors that Potently Reactivate Latent HIV. Viruses.

[27] Jones R.B., O’Connor R., Mueller S., Foley M.H., Szeto G., Karel D., Lichterfeld M., Kovacs C., Ostrowski M.A., Trocha A. (2014). Histone Deacetylase Inhibitors Impair the Elimination of HIV-Infected Cells by Cytotoxic T-Lymphocytes. PLoS Pathog..

[28] Clutton G., Xu Y., Baldoni P.L., Mollan K.R., Kirchherr J., Newhard W., Cox K., Kuruc J.D., Kashuba A., Barnard R. (2016). The Differential Short- and Long-Term Effects of HIV-1 Latency-Reversing Agents on T Cell Function. Sci. Rep..

[29] Walker-Sperling V.E., Pohlmeyer C.W., Tarwater P.M., Blankson J.N. (2016). The Effect of Latency Reversal Agents on Primary CD8+ T Cells: Implications for Shock and Kill Strategies for Human Immunodeficiency Vi-rus Eradication. EBioMedicine.

[30] Ogbomo H., Michaelis M., Kreuter J., Doerr H.W., Cinatl J. (2007). Histone Deacetylase Inhibitors Sup-press Natural Killer Cell Cytolytic Activity. FEBS Lett..

[31] Garrido C., Spivak A.M., Soriano-Sarabia N., Checkley M.A., Barker E., Karn J., Planelles V., Margolis D.M. (2016). HIV Latency-Reversing Agents Have Diverse Effects on Natural Killer Cell Function. Front. Immunol..

[32] Pace M., Williams J., Kurioka A., Gerry A.B., Jakobsen B., Klenerman P., Nwokolo N., Fox J., Fidler S., Frater J. (2016). Histone Deacetylase Inhibitors Enhance CD4 T Cell Susceptibility to NK Cell Killing but Re-duce NK Cell Function. PLoS Pathog..

[33] Ni L., Wang L., Yao C., Ni Z., Liu F., Gong C., Zhu X., Yan X., Watowich S.S., Lee D.A. (2017). The Histone Deacetylase Inhibitor Valproic Acid Inhibits NKG2D Expression in Natural Killer Cells through Sup-pression of STAT3 and HDAC3. Sci. Rep..

[34] Zhu S., Denman C.J., Cobanoglu Z.S., Kiany S., Lau C.C., Gottschalk S.M., Hughes D.P.M., Kleinerman E.S., Lee D. (2015). The Narrow-Spectrum HDAC Inhibitor Entinostat Enhances NKG2D Expression Without NK Cell Toxicity, Leading to Enhanced Recognition of Cancer Cells. Pharm. Res..

[35] Desimio M.G., Giuliani E., Ferraro A.S., Adorno G., Doria M. (2018). In Vitro Exposure to Prostratin but Not Bryostatin-1 Improves Natural Killer Cell Functions Including Killing of CD4(+) T Cells Harboring Reac-tivated Human Immunodeficiency Virus. Front. Immunol..

[36] Olesen R., Vigano S., Rasmussen T.A., Søgaard O., Ouyang Z., Buzon M.J., Bashirova A., Carrington M., Palmer S., Brinkmann C.R. (2015). Innate Immune Activity Correlates with CD4 T Cell-Associated HIV-1 DNA Decline during Latency-Reversing Treatment with Panobinostat. J. Virol..

[37] Garrido C., Tolstrup M., Søgaard O.S., Rasmussen T.A., Allard B., Soriano-Sarabia N., Archin N.M., Margolis D.M. (2019). In-vivo administration of histone deacetylase inhibitors does not impair natural killer cell function in HIV+ individuals. AIDS.

[38] Desimio M.G., Giuliani E., Doria M. (2017). The Histone Deacetylase Inhibitor SAHA Simultaneously Reac-tivates HIV-1 from Latency and Up-Regulates NKG2D Ligands Sensitizing for Natural Killer Cell Cytotoxicity. Virology.

[39] Garrido C., Abad-Fernandez M., Tuyishime M., Pollara J.J., Ferrari G., Soriano-Sarabia N., Margolis D.M. (2018). Interleukin-15-Stimulated Natural Killer Cells Clear HIV-1-Infected Cells following Latency Reversal Ex Vivo. J. Virol..

[40] Giuliani E., Desimio M.G., Doria M. (2019). Hexamethylene bisacetamide impairs NK cell-mediated clearance of acute T lymphoblastic leukemia cells and HIV-1-infected T cells that exit viral latency. Sci. Rep..

[41] Lanier L.L. (2005). NK CELL RECOGNITION. Annu. Rev. Immunol..

[42] Cerboni C., Neri F., Casartelli N., Zingoni A., Cosman D., Rossi P., Santoni A., Doria M. (2007). Human immunodeficiency virus 1 Nef protein downmodulates the ligands of the activating receptor NKG2D and inhibits natural killer cell-mediated cytotoxicity. J. Gen. Virol..

[43] Fogli M., Mavilio D., Brunetta E., Varchetta S., Ata K., Roby G., Kovacs C., Follmann D., Pende D., Ward J. (2008). Lysis of Endogenously Infected CD4+ T Cell Blasts by rIL-2 Activated Autologous Natural Killer Cells from HIV-Infected Viremic Individuals. PLoS Pathog..

[44] Ward J., Davis Z., Dehart J., Zimmerman E., Bosque A., Brunetta E., Mavilio D., Planelles V., Barker E. (2009). HIV-1 Vpr Triggers Natural Killer Cell–Mediated Lysis of Infected Cells through Activation of the ATR-Mediated DNA Damage Response. PLoS Pathog..

[45] Richard J., Sindhu S., Pham T.N.Q., Belzile J.-P., Cohen É.A. (2010). HIV-1 Vpr up-regulates expression of ligands for the activating NKG2D receptor and promotes NK cell–mediated killing. Blood.

[46] Matusali G., Tchidjou H.K., Pontrelli G., Bernardi S., D’Ettorre G., Vullo V., Buonomini A.R., Andreoni M., Santoni A., Cerboni C. (2013). Soluble ligands for the NKG2D receptor are released during HIV-1 infection and impair NKG2D expression and cytotoxicity of NK cells. FASEB J..

[47] Raulet D.H., Gasser S., Gowen B.G., Deng W., Jung H. (2013). Regulation of Ligands for the NKG2D Acti-vating Receptor. Annu. Rev. Immunol..

[48] Chretien A.S., Le Roy A., Vey N., Prebet T., Blaise D., Fauriat C., Olive D. (2014). Cancer-Induced Altera-tions of NK-Mediated Target Recognition: Current and Investigational Pharmacological Strategies Aiming at Restoring NK-Mediated Anti-Tumor Activity. Front. Immunol..

[49] Cifaldi L., Locatelli F., Marasco E., Moretta L., Pistoia V. (2017). Boosting Natural Killer Cell-Based Immu-notherapy with Anticancer Drugs: A Perspective. Trends Mol. Med..

[50] Zingoni A., Fionda C., Borrelli C., Cippitelli M., Santoni A., Soriani A. (2017). Natural Killer Cell Response to Chemotherapy-Stressed Cancer Cells: Role in Tumor Immunosurveillance. Front. Immunol..

[51] Desimio M.G., Covino D.A., Doria M. (2019). Potential of the NKG2D/NKG2DL Axis in NK Cell-Mediated Clearance of the HIV-1 Reservoir. Int. J. Mol. Sci..

[52] Tsai A., Irrinki A., Kaur J., Cihlar T., Kukolj G., Sloan D.D., Murry J.P. (2017). Toll-Like Receptor 7 Agonist GS-9620 Induces HIV Expression and HIV-Specific Immunity in Cells from HIV-Infected Individuals on Suppressive Antiretroviral Therapy. J. Virol..

[53] Macedo A.B., Novis C.L., De Assis C.M., Sorensen E.S., Moszczynski P., Huang S.-H., Ren Y., Spivak A.M., Jones R.B., Planelles V. (2018). Dual TLR2 and TLR7 agonists as HIV latency-reversing agents. JCI Insight.

[54] Lim S.-Y., Osuna C.E., Hraber P.T., Hesselgesser J., Gerold J.M., Barnes T.L., Sanisetty S., Seaman M.S., Lewis M.G., Geleziunas R. (2018). TLR7 agonists induce transient viremia and reduce the viral reservoir in SIV-infected rhesus macaques on antiretroviral therapy. Sci. Transl. Med..

[55] Bam R.A., Hansen D., Irrinki A., Mulato A., Jones G.S., Hesselgesser J., Frey C.R., Cihlar T., Yant S.R. (2017). TLR7 Agonist GS-9620 Is a Potent Inhibitor of Acute HIV-1 Infection in Human Peripheral Blood Mononuclear Cells. Antimicrob. Agents Chemother..

[56] Wei D.G., Chiang V., Fyne E., Balakrishnan M., Barnes T., Graupe M., Hesselgesser J., Irrinki A., Murry J., Stepan G. (2014). Histone Deacetylase Inhibitor Romidepsin Induces HIV Expression in CD4 T Cells from Patients on Suppressive Antiretroviral Therapy at Concentrations Achieved by Clinical Dosing. PLoS Pathog..

[57] Darcis G., Kula A., Bouchat S., Fujinaga K., Corazza F., Ait-Ammar A., Delacourt N., Melard A., Kabeya K., Vanhulle C. (2015). An in-Depth Comparison of Latency-Reversing Agent Combinations in various in Vitro and Ex Vivo HIV-1 Latency Models Identified Bryostatin-1+JQ1 and Ingenol-B+JQ1 to Potently Reac-tivate Viral Gene Expression. PLoS Pathog..

[58] Bonet M.M., Clemente M.I., Serramía M.J., Muñoz E., Moreno S., Muñoz-Fernández M. (2015). Ángeles Synergistic Activation of Latent HIV-1 Expression by Novel Histone Deacetylase Inhibitors and Bryostatin-1. Sci. Rep..

[59] Valés-Gómez M., Chisholm S.E., Cassady-Cain R.L., Roda-Navarro P., Reyburn H.T. (2008). Selective In-duction of Expression of a Ligand for the NKG2D Receptor by Proteasome Inhibitors. Cancer Res..

[60] Zhao M., De Crignis E., Rokx C., Verbon A., van Gelder T., Mahmoudi T., Katsikis P.D., Mueller Y.M. (2019). T cell toxicity of HIV latency reversing agents. Pharmacol. Res..

[61] Iwata S., Yano S., Ito Y., Ushijima Y., Gotoh K., Kawada J.-I., Fujiwara S., Sugimoto K., Isobe Y., Nishiyama Y. (2011). Bortezomib induces apoptosis in T lymphoma cells and natural killer lymphoma cells independent of Epstein-Barr virus infection. Int. J. Cancer.

[62] Moreau P., Karamanesht I.I., Domnikova N., Kyselyova M.Y., Vilchevska K.V., Doronin V.A., Schmidt A., Hulin C., Leleu X., Esseltine D.L. (2012). Pharmacokinetic, Pharmacodynamic and Covariate Analysis of Subcutaneous Versus Intravenous Administration of Bortezomib in Patients with Relapsed Multi-ple Myeloma. Clin. Pharmacokinet..

[63] Keedy K.S., Archin N.M., Gates A.T., Espeseth A., Hazuda D.J., Margolis D.M. (2009). A Limited Group of Class I Histone Deacetylases Acts To Repress Human Immunodeficiency Virus Type 1 Expression. J. Virol..

[64] Singh A.K., Bishayee A., Pandey A.K. (2018). Targeting Histone Deacetylases with Natural and Synthetic Agents: An Emerging Anticancer Strategy. Nutrients.

[65] Zaikos T.D., Painter M.M., Kettinger N.T.S., Terry V.H., Collins K.L. (2018). Class 1-Selective Histone Deacetylase (HDAC) Inhibitors Enhance HIV Latency Reversal while Preserving the Activity of HDAC Isoforms Necessary for Maximal HIV Gene Expression. J. Virol..

[66] Pérez M., de Vinuesa A.G., Sanchez-Duffhues G., Marquez N., Bellido M.L., Muñoz-Fernandez M.A., Moreno S., Castor T.P., Calzado M.A., Muñoz E. (2010). Bryostatin-1 Synergizes with Histone Deacetylase In-hibitors to Reactivate HIV-1 from Latency. Curr. HIV. Res..

[67] Exposito J.G., Luque-Ballesteros L., Navarro J., Curran A., Burgos J., Ribera E., Torrella A., Planas B., Badía R., Martin-Castillo M. (2019). Latency reversal agents affect differently the latent reservoir present in distinct CD4+ T subpopulations. PLoS Pathog..

[68] Bali P., Pranpat M., Bradner J., Balasis M., Fiskus W., Guo F., Rocha K., Kumaraswamy S., Bo-yapalle S., Atadja P. (2005). Inhibition of Histone Deacetylase 6 Acetylates and Disrupts the Chaperone Function of Heat Shock Protein 90: A Novel Basis for Antileukemia Activity of Histone Deacetylase Inhibitors. J. Biol. Chem..

[69] Anderson I., Low J.S., Weston S., Weinberger M., Zhyvoloup A., Labokha A.A., Corazza G., Kitson R.A., Moody C., Marcello A. (2014). Heat shock protein 90 controls HIV-1 reactivation from latency. Proc. Natl. Acad. Sci. USA.

[70] Dominguez-Villar M., Gautron A.-S., De Marcken M., Keller M.J., Hafler D.A. (2015). TLR7 induces anergy in human CD4+ T cells. Nat. Immunol..

[71] Hokello J., Lakhikumar Sharma A., Tyagi M. (2021). AP-1 and NF-κB Synergize to Transcriptionally Acti-vate Latent HIV upon T-Cell Receptor Activation. FEBS Lett..

[72] Zhao S., Wang H., Nie Y., Mi Q., Chen X., Hou Y. (2012). Midkine Upregulates MICA/B Expression in Hu-man Gastric Cancer Cells and Decreases Natural Killer Cell Cytotoxicity. Cancer Immunol. Immunother..

[73] Cary D.C., Peterlin B.M. (2020). Proteasomal Inhibition Potentiates Latent HIV Reactivation. AIDS Res. Hum. Retroviruses.

[74] Shan L., Deng K., Shroff N.S., Durand C.M., Rabi S.A., Yang H.-C., Zhang H., Margolick J.B., Blankson J.N., Siliciano R.F. (2012). Stimulation of HIV-1-Specific Cytolytic T Lymphocytes Facilitates Elimination of Latent Viral Reservoir after Virus Reactivation. Immun..

[75] Banga R., Procopio F.A., Cavassini M., Perreau M. (2016). In Vitro Reactivation of Replication-Competent and Infectious HIV-1 by Histone Deacetylase Inhibitors. J. Virol..

[76] Blazkova J., Chun T.W., Belay B.W., Murray D., Justement J.S., Funk E.K., Nelson A., Hallahan C.W., Moir S., Wender P.A. (2012). Effect of Histone Deacetylase Inhibitors on HIV Production in Latently In-fected, Resting CD4(+) T Cells from Infected Individuals Receiving Effective Antiretroviral Therapy. J. Infect. Dis..

[77] Beans E.J., Fournogerakis D., Gauntlett C., Heumann L.V., Kramer R., Marsden M.D., Murray D., Chun T.-W., Zack J.A., Wender P.A. (2013). Highly potent, synthetically accessible prostratin analogs induce latent HIV expression in vitro and ex vivo. Proc. Natl. Acad. Sci. USA.

[78] Marsden M.D., Loy B.A., Wu X., Ramirez C.M., Schrier A.J., Murray D., Shimizu A., Ryckbosch S.M., Near K.E., Chun T.-W. (2017). In vivo activation of latent HIV with a synthetic bryostatin analog effects both latent cell “kick” and “kill” in strategy for virus eradication. PLoS Pathog..

[79] Yang H., Li X., Yang X., Lu P., Wang Y., Jiang Z., Pan H., Zhao L., Zhu Y., Khan I.U. (2019). Dual effects of the novel ingenol derivatives on the acute and latent HIV-1 infections. Antivir. Res..

[80] Marsden M.D., Wu X., Navab S.M., Loy B.A., Schrier A.J., DeChristopher B.A., Shimizu A.J., Hardman C.T., Ho S., Ramirez C. (2018). Characterization of designed, synthetically accessible bryostatin analog HIV latency reversing agents. Virol..

[81] Pardons M., Fromentin R., Pagliuzza A., Routy J.-P., Chomont N. (2019). Latency-Reversing Agents Induce Differential Responses in Distinct Memory CD4 T Cell Subsets in Individuals on Antiretroviral Therapy. Cell Rep..

