# Supplementary material for: Combinations of Histone Deacetylase Inhibitors with Distinct Latency Reversing Agents Variably Affect HIV Reactivation and Susceptibility to NK Cell-Mediated Killing of T Cells That Exit Viral Latency
Source: Int J Mol Sci. 2021 Jun 22;22(13):6654. doi: 10.3390/ijms22136654 (PMC8267728; doi:10.3390/ijms22136654)
Supplement: Supplementary file 1 [file ijms-22-06654-s001.zip › ijms-1274545-Supplementary.pdf]

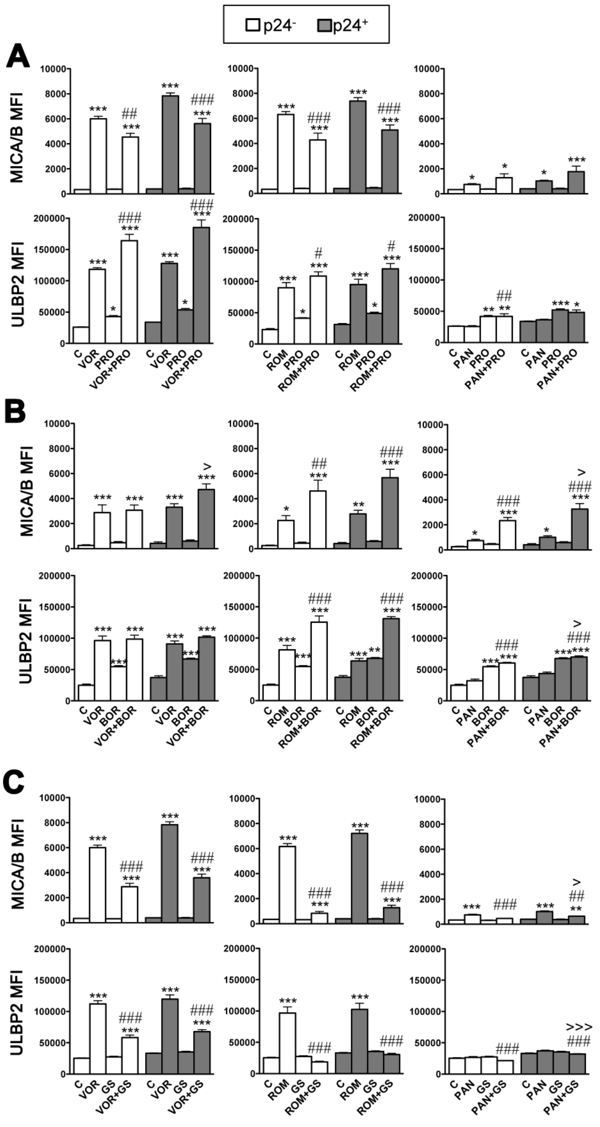

Supplementary Fig. S1. Modulation of NKG2DLs expression by single and two-drug combinations in J1.1 cells. J1.1 cells were treated for 48 h with solvent as control (CTR), 10 μM VOR, 20 nM ROM, 20 nM PAN, 1 μM PRO, 5 nM BOR, or 3 μM GS-9620 (GS) used alone or combining each HDACi (VOR, ROM, PAN) with PRO, BOR, or GS. Mean fluorescence intensity (MFI) of MICA/B and ULBP2 expression (mean ± SEM MFI) was quantified on both p24$^-$ and p24$^+$ cells after exposure to single or combined HDACi/PRO (A), HDACi/BOR (B), and HDACi/GS (C). Bars represent mean ± SEM (n=7). Statistics was performed using One-way Anova with Bonferroni post test to perform multiple comparisons. Versus control (CTR): **P* < 0.05, ***P* < 0.01, ****P* < 0.001. Versus HDACi alone: $^{\#}P < 0.05$, $^{\#\#}P < 0.01$, $^{\#\#\#}P < 0.001$. Versus p24$^-$ cells: $^{>}P < 0.05$, $^{>>>}P < 0.001$.
